# In Situ Encapsulation of Graphene Quantum Dots in Highly Stable Porphyrin Metal-Organic Frameworks for Efficient Photocatalytic CO_2_ Reduction

**DOI:** 10.3390/molecules28124703

**Published:** 2023-06-12

**Authors:** Qin Yu, Xusheng Wang, Wenbin Wu, Xinya Feng, Deyu Kong, Usman Khan, Xiaohui Ren, Lan Li

**Affiliations:** 1 Institute of Functional Porous Materials, School of Materials Science and Engineering, Zhejiang Sci-Tech University, Hangzhou 310018, China; 2021316101103@mails.zstu.edu.cn (Q.Y.); 2022316101108@mails.zstu.edu.cn (W.W.); 2022316101030@mails.zstu.edu.cn (X.F.); 2022316101070@mails.zstu.edu.cn (D.K.); usman@zstu.edu.cn (U.K.); 2 Guangdong Provincial Key Laboratory of Functional Supramolecular Coordination Materials and Applications, Jinan University, Guangzhou 510632, China; 3 Tongxiang Research Institute, Zhejiang Sci-Tech University, Jiaxing 314500, China; 4 Zhejiang LINIX Motor Co., Ltd., Jinhua 322118, China; 5 The State Key Laboratory of Refractories and Metallurgy, School of Materials and Metallurgy, Wuhan University of Science and Technology, Wuhan 430081, China; xhren@wust.edu.cn; 6 College of Materials and Chemistry, China Jiliang University, Hangzhou 310018, China; lanli@cjlu.edu.cn

**Keywords:** metal-organic frameworks, porous materials, graphene quantum dots, photocatalyst, photocatalysis, photoreduction, CO_2_, PCN-222, porphyrin, MOFs

## Abstract

Photocatalytic CO_2_ reduction to valuable hydrocarbon solar fuel is of great significance but still challenging. Strong CO_2_ enrichment ability and easily adjustable structures make metal-organic frameworks (MOFs) potential photocatalysts for CO_2_ conversion. Even though pure MOFs have the potential for photoreduction of CO_2_, the efficiency is still quite low due to rapid photogenerated electron–hole recombination and other drawbacks. In this work, graphene quantum dots (GQDs) were in situ encapsulated into highly stable MOFs via a solvothermal method for this challenging task. The GQDs@PCN-222 with encapsulated GQDs showed similar Powder X-ray Diffraction (PXRD) patterns to PCN-222, indicating the retained structure. The porous structure was also retained with a Brunauer–Emmett–Teller (BET) surface area of 2066 m^2^/g. After incorporation of GQDs, the shape of GQDs@PCN-222 particles remained, as revealed by the scanning electron microscope (SEM). As most of the GQDs were covered by thick PCN-222, it was hard to observe those GQDs using a transmission electron microscope (TEM) and a high-resolution transmission electron microscope (HRTEM) directly, the treatment of digested GQDs@PCN-222 particles by immersion in a 1 mM aqueous KOH solution can make the incorporated GQDs visible in TEM and HRTEM. The linker, deep purple porphyrins, make MOFs a highly visible light harvester up to 800 nm. The introduction of GQDs inside PCN-222 can effectively promote the spatial separation of the photogenerated electron–hole pairs during the photocatalytic process, which was proved by the transient photocurrent plot and photoluminescence emission spectra. Compared with pure PCN-222, the obtained GQDs@PCN-222 displayed dramatically enhanced CO production derived from CO_2_ photoreduction with 147.8 μmol/g/h in a 10 h period under visible light irradiation with triethanolamine (TEOA) as a sacrificial agent. This study demonstrated that the combination of GQDs and high light absorption MOFs provides a new platform for photocatalytic CO_2_ reduction.

## 1. Introduction

The excessive emission of CO_2_ is the major contributor to global warming [1,2,3]. Capture and conversion of CO_2_ to value-added chemicals is a major issue for scientists all around the world [4,5,6]. Photocatalysis is an energy-saving and highly efficient technology [7,8,9,10,11,12,13,14,15,16,17]. As a kind of photocatalytic technology, photocatalytic reduction of CO_2_ to valuable chemicals is of great significance but still challenging [18,19,20,21,22]. So far, several photocatalysts have been devoted to photocatalytic CO_2_ reduction, but are still unsatisfied due to low adsorption of CO_2_, narrow and weak light absorption, and rapid recombination of the photogenerated electron–hole pairs [6,23,24]. Searching new kinds of photocatalysts for photocatalytic CO_2_ reduction under visible light region is still vital and challenging work.

Metal-organic frameworks (MOFs), assembled by inorganic metal ions or clusters and organic ligands through coordination bonds, are a new kind of material with high porosity, high specific surface area, and easily adjustable structures [25,26,27,28,29,30]. With those excellent properties, MOFs had been widely used in many fields, such as gas adsorption and separation, drug loading, catalysis and so on [31,32,33,34,35,36,37,38]. Especially, strong CO_2_ enrichment ability makes MOFs to be potential photocatalysts for CO_2_ conversion [39]. In addition, the photocatalytic conversion of CO_2_ by MOFs can be promoted by adjusting the catalytic active center, light absorption range and energy bandgap position, etc. [40,41,42]. Benefitting from those advantages, MOFs had been studied for the photocatalytic conversion of CO_2_ directly [43,44,45,46,47,48]. For example, NH_2_-MIL-125(Ti) had been used for photoreduction of CO_2_ under visible light irradiation by the Li group, with produced formic acid reaching 16 μmol/g/h [47]. Afterwards, with highly visible light harvesting ability, porphyrin-involved MOFs, PCN-222, had also been applied for selective capture and further photoreduction of CO_2_, with activity reaching 60 μmol/g/h [48]. Even though pure MOFs have the potential for photoreduction of CO_2_, the efficiency is still quite low due to rapid photogenerated electron–hole recombination and other drawbacks. To solve those problems, components, such as platinum, gold, silver, C_3_N_4_, graphene, and Zn_2_GeO_4_, had been combined with MOFs to improve the photocatalytic efficiency, but it is still hard to satisfy the high requirements [49,50,51,52,53].

Recently, graphene quantum dots (GQDs) became a rising star in carbon nanomaterials due to their excellent light stability, biocompatibility, water solubility and low toxicity [54]. The diameter of GQDs is often less than 10 nm, mainly composed of sp^2^ amorphous carbon and sp^2^ graphite carbon. In particular, the GQDs can absorb light from ultraviolet to near-infrared, and have good luminescence and electron conduction ability. In addition, the GQDs can be used as electronic sinks, making them potential photocatalytic cocatalysts. For example, Kang et al. reported GQDs can be combined with carbon nitride to form graphene quantum dots–carbon nitride nanocomposite for photocatalytic solar water splitting with overall solar energy conversion efficiency of 2.0% [55].

In order to solve the problem of low activity of photocatalytic reduction of CO_2_ by pure MOFs, herein, GQDs had been in situ encapsulated into a highly stable porphyrin MOF, PCN-222, obtaining GQDs@PCN-222. The GQDs@PCN-222 can combine the advantages of MOFs and GQDs, overcome the disadvantage of low catalytic activity of pure MOFs, and be used for high-efficiency photocatalytic CO_2_ reduction.

## 2. Results and Discussion

### 2.1. Synthesis and Characterization of GQDs

GQDs were prepared by electrolysis of graphite electrodes following the reported method [55,56]. Typically, 500 mL of deionized water was added into an 800 mL beaker and two high-purity graphite electrodes were put into the above beaker. The distance between the two electrodes was 7 cm in the beaker, and the potential was set as 30 V DC. The beaker was covered by a plastic film to prevent the evaporation of water and the contamination of dust during the electrolysis process. Noting that hydrogen and oxygen gas will be produced during the electrolysis process, some small holes should be punched on the cover. For safety, the equipment should be placed into a fume hood in case of the accumulation of explosive hydrogen. The clear and colorless water gradually turned black during the two weeks of electrolysis. Afterwards, the black electrolyte was centrifuged for one hour with a speed of 20,000 rpm to remove the large particles, and the supernatant was then evaporated to obtain the desired GQDs.

**Figure 1:**
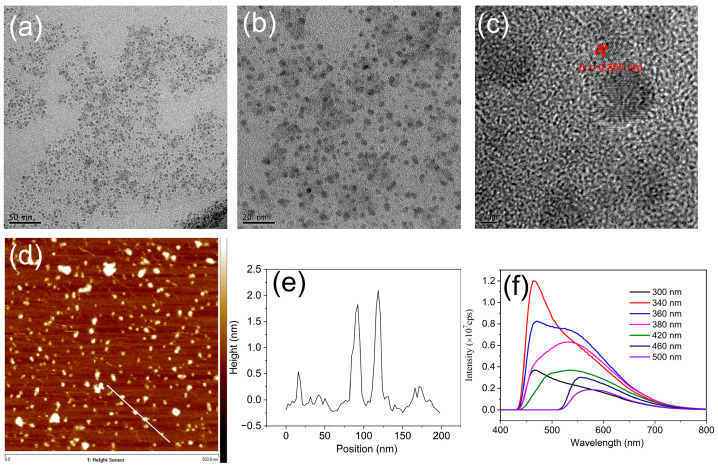
Characterizations of GQDs prepared by electrolysis of graphite electrodes. (**a**,**b**) TEM and (**c**) HRTEM of GQDs with a scale bar of 50 nm, 20 nm, and 2 nm, respectively; (**d**) AFM of GQDs; (**e**) The height of selected GQDs in (**d**); (**f**) PL emission spectra of GQDs aqueous dispersion at different excitation wavelengths (300 nm to 500 nm).

The transmission electron microscope (TEM) and the high-resolution transmission electron microscope (HRTEM) gave a visual method to measure the size and morphology of GQDs. As shown in Figure 1a,b pictured by TEM, ultrafine GQDs were obtained with a size of around 3 nm and the size distributions were quite uniform. HRTEM (Figure 1c) further discovered that the GQDs with high crystallinity and a 0.207 nm lattice spacing of graphene (101) are identified. The atomic force microscope (AFM) is an effective piece of equipment to measure the thickness of GQDs. As shown in Figure 1d,e, the thickness of the prepared GQDs was about 2.0 nm and the corresponding layer number is about 5. Interestingly, a small amount of GQDs can even reach a single layer, and the thickness is only about 0.34 nm. The photoluminescence (PL) emission properties of GQDs were further studied with a fluorescence spectrometer. The PL emission spectra showed a typical excitation-wavelength dependent behavior of GQDs: shifting the excitation wavelength from λ = 300 to 500 nm, the corresponding maximum of emission spectra are shifted from λ = 466 to 577 nm (Figure 1f). This phenomenon is similar to the previously reported results [55,56]. While the PL emission in GQDs is not well understood, the prevailing view is that emission results from radiative recombination at surface confined defect states. The excitation-wavelength-dependent behavior is thought to be due to the heterogeneity of such emission states in the sample from either multiple emission sites on each dot or different emissive sites between individual dots.

### 2.2. Synthesis and Characterization of PCN-222 and GQDs@PCN-222

**Figure 2:**
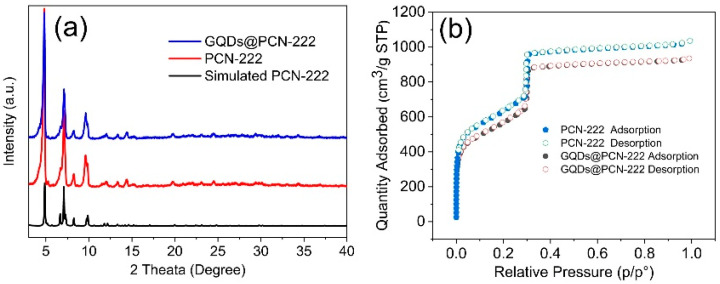
Structural characterization of PCN-222 and GQDs@PCN-222. (**a**) PXRD patterns of simulated PCN-222, PCN-222, and GQDs@PCN-222; (**b**) N_2_ sorption isotherms of PCN-222, and GQDs@PCN-222.

The synthesis and activation of pure PCN-222 followed the reported literature [48]. The successful synthesis of PCN-222 was proved by Powder X-Ray Diffraction (PXRD), which showed almost identical patterns to the simulated one (Figure 2a). N_2_ sorption isotherm is an effective way to characterize the porous structure of photocatalysts. As shown in Figure 2b, both PCN-222 have a completely reversible Type IVb isotherm according to the classifications of IUPAC, which indicates that mesopores with pore width lower than 4 nm are existing [57]. The pore size distribution of PCN-222 showed that the biggest pore width of PCN-222 is around 3.7 nm (Figure S1), which is similar to the previously reported results. The average pore size of PCN-222 is around 2.3 nm. In addition, a really high Brunauer–Emmett–Teller (BET) surface area of 2339 m^2^/g was also obtained by the N_2_ sorption isotherm (Figure 2b). As the size of GQDs is smaller than the pore of PCN-222, those GQDs might be enclosed within the framework pores of PCN-222. The GQDs were in situ encapsulated into the MOFs by solvothermal method to obtain GQDs@PCN-222. The GQDs@PCN-222 with encapsulated GQDs showed similar PXRD patterns to PCN-222, indicating the retained crystal structure. The porous structure was also retained with a high BET surface area of 2066 m^2^/g. Notably, the pore size distribution of GQDs@PCN-222 had no obvious difference from PCN-222 with the biggest pore width of around 3.7 nm. The average pore size of GQDs@PCN-222 is also similar to PCN-222 with a value of 2.3 nm.

**Figure 3:**
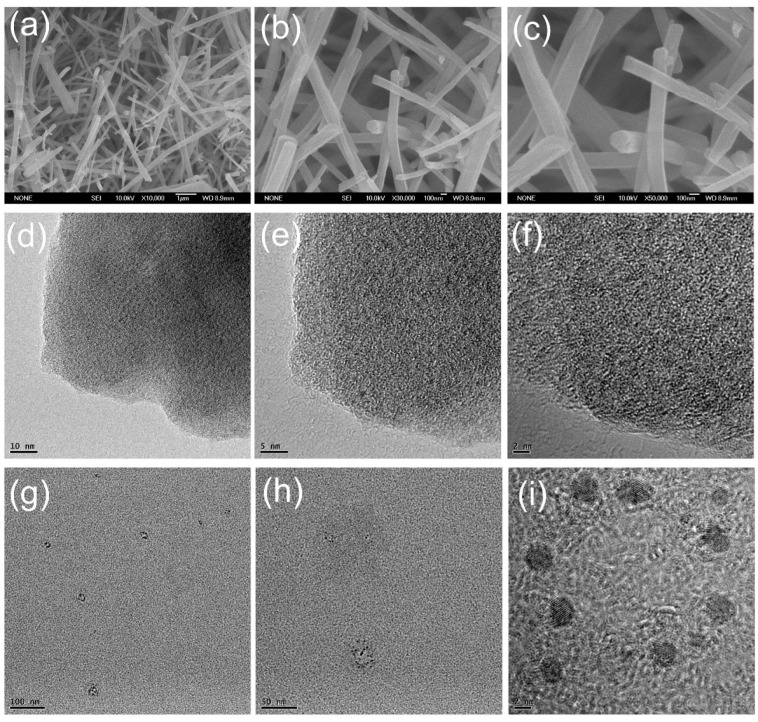
Electron microscope images of GQDs@PCN-222, and the digested GQDs@PCN-222. (**a**–**c**) SEM images of GQDs@PCN-222 with a scale bar of 1 μm, 100 nm, 100 nm, respectively; (**d**–**f**) TEM and HRTEM images of GQDs@PCN-222 with a scale bar of 10 nm, 5 nm, 2 nm, respectively; (**g**–**i**) TEM and HRTEM images of the digested GQDs@PCN-222 by KOH solution with a scale bar of 100 nm, 50 nm, 2 nm, respectively.

The scanning electron microscope (SEM) images showed that all the pure PCN-222 particles had a nanorod shape and a diameter of around 500 nm (Figure S2). After the incorporation of GQDs, the shape of GQDs@PCN-222 rods was retained but the diameter was reduced to 150 nm (Figure 3a–c). The decreased diameter of MOFs might be ascribed to the existence of carboxylate groups on the surface of incorporated GQDs. As most of the GQDs were covered by thick PCN-222, it was hard to observe those GQDs using high-resolution transmission electron microscopy (HRTEM) directly (Figure 3d–f) [58]. Comparatively, GQDs can be observed on the surface of GQDs/PCN-222 as GQDs were not covered by MOFs (Figure S3). For the more thorough characterization of the GQDs particles incorporated in GQDs@PCN-222 rods, the MOF composite sample was digested by immersing it in a 1 mM aqueous KOH solution. This treatment resulted in the dissolution of the GQDs@PCN-222 rods to yield a suspension of the GQDs because PCN-222 is not stable while GQDs are stable in the high alkali condition. GQDs were then obtained by filtration and extraction of the suspension of the GQDs with diethyl ether. HRTEM of these GQDs (Figure 3g–i) revealed an average size of the GQDs of approximately 3 nm, with a rather narrow size distribution.

### 2.3. Photocatalytic Properties

**Figure 4:**
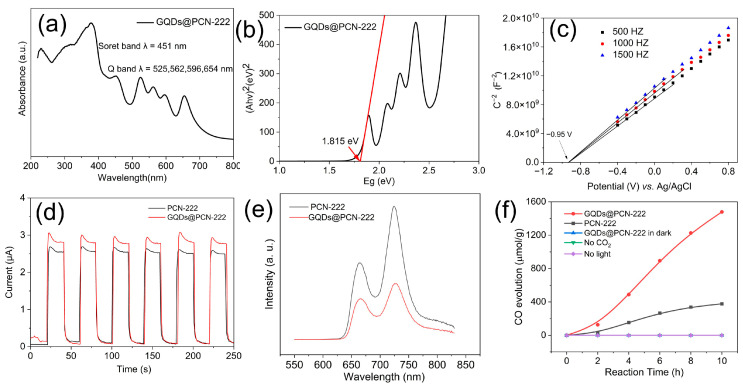
Photocatalytic performance and the photocatalytic mechanism-related characterizations. (**a**) Solid UV-vis diffuse reflectance spectrum of GQDs@PCN-222; (**b**) Tauc plot of GQDs@PCN-222; (**c**) Mott-Schottky plots of GQDs@PCN-222 with a frequency of 500, 1000, and 1500 HZ; (**d**) Photocurrent and (**e**) PL emission with excitation wavelength at 365 nm of PCN-222 and GQDs@PCN-222; (**f**) Photocatalytic CO_2_ reduction by photocatalysts.

The solid UV-Vis diffuse reflectance spectrum (UV-Vis DRS) showed that the absorption range of organic ligand meso-tetra(4-carboxyphenyl)porphine (H_2_TCPP) can reach up to 800 nm with peaks of Soret band at 355 nm and Q bands at 514, 553, 591, and 646 nm (Figure S4a). Correspondingly, a similar light absorption range and peaks (Soret band at 449 nm and Q bands at 520, 560, 595, and 652 nm) were obtained for PCN-222 synthesized by a solvothermal method involving H_2_TCPP (Figure S5a). Notably, after the encapsulation of GQDs in MOFs, no obvious change of absorption peaks (Soret band at 451 nm and Q bands at 525, 562, 596, 654 nm) and light harvesting range can be detected (Figure 4a). Even though the absorbance range and peaks of GQDs@PCN-222 did not change much after encapsulation of GQDs, the photocurrent had increased significantly (Figure 4d), and the PL emission intensity decreased obviously (Figure 4e), which indicated that the introduction of GQDs improves the separation efficiency and decreases the recombination of photogenerated electron–hole pairs of the catalyst. The bandgap of ligand H_2_TCPP was calculated from the Tauc plot to be 1.75 eV (Figure S4b), while the bandgaps of PCN-222 and GQDs@PCN-222 were calculated to be 1.79 eV and 1.82 eV, respectively (Figure 4b and Figure S5b) [59,60]. The above results indicated that H_2_TCPP, PCN-222, and GQDs@PCN-222 have similar values of bandgap. The conduction band (CB) minimums of PCN-222 and GQDs@PCN-222 were also measured by Mott–Schottky curves to be −0.9 eV and −0.95 eV, respectively (Figure 4c and Figure S6). Accordingly, the valence band (VB) maximums of PCN-222 and GQDs@PCN-222 were calculated to be 0.89 eV and 0.87 eV, respectively. Therefore, both PCN-222 and GQDs@PCN-222 are thermodynamics favorable for photocatalytic CO_2_ reduction with triethanolamine (TEOA) as a sacrificial agent.

**Figure 5:**
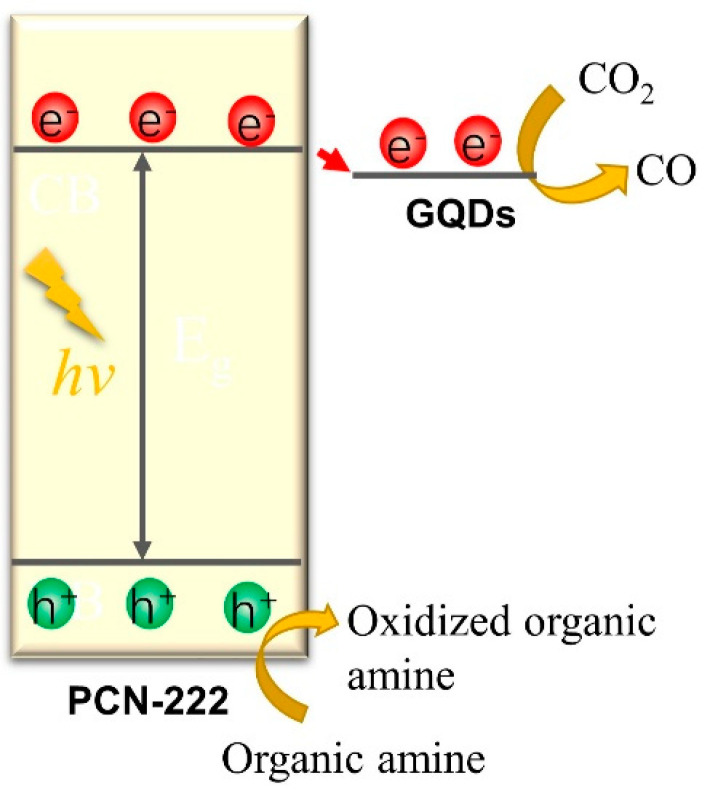
Proposed photocatalytic mechanism of CO_2_ reduction to CO by GQDs@PCN-222.

In view of the advantages of GQDs@PCN-222 rods, it was then used for visible light photocatalytic CO_2_ reduction. GQDs@PCN-222 rods were evenly dispersed on the quartz filter membrane, which had been placed in the solid-gas photocatalytic reaction device. A mixture of acetonitrile and triethanolamine (the volume ratio of acetonitrile to TEOA is 4:1) was then added into the photocatalytic reaction device, too. After three instances of vacuuming and CO_2_ filling (making sure that all the air in the system was replaced by CO_2_), the reaction system was balanced in dark for 1 h and then the photoreduction reaction of CO_2_ started after visible light irradiation (300 W Xe lamp with 420 nm cut-off filter). The product was measured by gas chromatography with both TCD and FID detectors every 2 h. As shown in Figure 4f, the produced CO of GQDs@PCN-222 increased steadily under visible light irradiation, and its activity reached 1 500 μmol/g after 10 h. No hydrogen gas was detected during the whole photocatalytic process. Relative to the PCN-222 (380 μmol/g), the activity of GQDs@PCN-222 increased nearly four times. At the same time, the comparative experiment showed that no CO was produced without GQDs@PCN-222. In addition, it has no catalytic activity when there is no CO_2_ or light. Combined with the above photocurrent and PL emission data, we speculate that the introduction of GQDs can greatly improve the separation of photogenerated carriers of PCN-222, and then improve the visible light photocatalytic carbon dioxide reduction activity. The enhanced activity of GQDs@PCN-222 than PCN-222 is because the GQDs can be used as an electronic sink, making them potential photocatalytic cocatalysts to accumulate the photogenerated electrons from PCN-222. A comparison of photocatalysts for CO_2_ reduction with previous works had also been summarized in Table S1, which showed that GQDs@PCN-222 is an outstanding photocatalyst for CO_2_ reduction. The proposed photocatalytic reduction mechanism of CO_2_ to CO by GQDs@PCN-222 can be illustrated as follows (Figure 5). Under visible light irradiation, photogenerated electrons were hopped from VB to CB of PCN-222 and then transferred to GQDs covered by MOF. The electrons will be accumulated on GQDs and reduce the adsorbed CO_2_ to CO. The residual holes on PCN-222 will oxidize the organic amine (TEOA) to oxidized organic amine. The overall reduction process of CO_2_ in the form of photochemical reactions was also shown (Equations (1)–(3)). Powder X-ray diffraction (PXRD) of GQDs@PCN-222 before and after photocatalysis showed that the photocatalyst still retained its original structure after photocatalysis (Figure S7).

(1) $$\mathrm{GQDs}\text{@}\mathrm{PCN}\text{-}222\;\overset{hv}{\to }e^{-}(\mathrm{GQDs})+h^{+}(\mathrm{PCN}\text{-}222)$$

2e^−^(GQDs) + CO_2_ + 2H^+^ → CO + H_2_O (2)

h^+^(PCN-222) + TEOA → PCN-222 + TEOA^+^ (3)

## 3. Materials and Methods

### 3.1. Chemical Regents

High-purity graphite electrodes (99.9995%, metal basis, Alfa Aesar, Heysham, Britain), *N*,*N*-diethylformamide (DEF, 99%, Adamas, Shanghai, China), Zirconium(IV) Chloride (ZrCl_4_, 98%, Adamas, Shanghai, China), Meso-tetra(4-carboxyphenyl)porphine (H_2_TCPP, 98%, TCI, Tokyo, Japan), Benzoic acid (99%, Adamas, Shanghai, China), *N*,*N*-Dimethylformamide (DMF, 99.5%, Adamas, Shanghai, China), Hydrochloric Acid (HCl, 4 M, 37%, Adamas, Shanghai, China) acetone (99.5%, Adamas, Shanghai, China), Sodium Sulfate (Na_2_SO_4_, 99%, Greagent, Shanghai, China), Ethanol (99.5%, Adamas, Shanghai, China), Nafion ‘D-521 dispersion, 5% *w*/*w* in water and 1-propanol (Alfa Aesar, Heysham, Britain), Triethanolamine (TEOA, 97%, Merk, Darmstadt, Germany), Diethyl ether (99.7%, Greagent, Shanghai, China).

### 3.2. The Synthesis of Graphene Quantum Dots (GQDs)

A total of 500 mL of deionized water was added into a volume of 800 mL beaker and then two high-purity graphite electrodes were put into the above beaker. In the beaker, the distance between two electrodes was set to 7 cm, and the potential was set to 30 V DC. After electrolysis of the two high-purity graphite electrodes for two weeks, the clear and colorless pure water gradually turned black. The black electrolyte was then centrifuged for one hour with a speed of 20,000 rpm, and the supernatant was then evaporated to obtain the required GQDs.

### 3.3. Preparation of GQDs Dispersion

A total of 25 mg of GQDs were dispersed into 5 mL of *N*,*N*-diethylformamide (DEF), resulting in 5 mg/mL of GQDs dispersion.

### 3.4. Synthesis of PCN-222

For the synthesis of PCN-222, a mixture of ZrCl_4_ (70 mg), meso-tetra(4-carboxyphenyl)porphine (H_2_TCPP, 50 mg), and benzoic acid (2700 mg) was ultrasonically dissolved in 8 mL of DEF in a volume of 20 mL Teflon-lined bomb. The mixture was heated in a 120 °C oven for 48 h and then a 130 °C oven for 24 h. After cooling down to room temperature, the sample was collected by centrifugation and washed with DMF three times. To further activate the sample, the obtained PCN-222 was suspended in a solution of 1.5 mL of 4 M HCl in 100 mL of DMF. Then, the mixture was heated in a 120 °C oven for 12 h. The sample was then centrifuged and washed with acetone three times. Finally, the sample was dried in a vacuum overnight at 80 °C, and about 60 mg of PCN-222 was obtained.

### 3.5. Synthesis of GQDs@PCN-222

For the synthesis of the GQDs@PCN-222, a mixture of ZrCl_4_ (70 mg), H_2_TCPP (50 mg), and benzoic acid (2700 mg) was ultrasonically dissolved in 8 mL of DEF in a 20 mL volume of Teflon-lined bomb. Then, 0.24 mL of GQDs dispersion (5 mg/mL) was added. The mixture was heated in a 120 °C oven for 48 h and then a 130 °C oven for 24 h. After cooling down to room temperature, the sample was collected by centrifugation and washed with DMF three times. To further activate the sample, the obtained PCN-222 was suspended in a solution of 1.5 mL of 4 M HCl in 100 mL of DMF. Then, the mixture was heated in a 120 °C oven for 12 h. The sample was then centrifuged and washed with acetone three times. Lastly, the sample was dried in a vacuum overnight at 80 °C.

### 3.6. Characterizations

The powder X-ray diffraction (PXRD) measurements were recorded on a powder X-ray diffractometer (MiniFlex 600, Rigaku Corporation, Tokyo, Japan) with Cu Ka radiation. X-ray photoelectron spectroscopy (XPS) data were acquired from a Thermo Fisher Scientific K-Alpha electron spectrometer (ESCALAB 250Xi, Thermo Fisher Scientific, Waltham, MA, USA). The morphologies of the samples were characterized by the scanning electron microscopy (SEM, JSM6700-F, JEOL, Akishima, Japan) and high-resolution transmission electron microscopy (HRTEM, Tecnai F20, FEI, Hillsboro, OR, USA). The specific surface areas were measured by Brunauer–Emmett–Teller (BET) on a Micrometrics ASAP 2020 system (ASAP 2020, Micrometrics Instrument Corp., Norcross, GA, USA). The samples were degassed at 120 °C for 2 h before the measurement and the N_2_ sorption isotherms were obtained at 77 K. The PL emission spectra were recorded on a Horiba FluoroLog-3 spectrometer (FluoroLog-3, Horiba, Ltd., Kyoto, Japan). The solid UV-Vis absorption spectra were conducted using a UV–Vis spectrophotometer (UH4150, Hitachi, Tokyo, Japan).

### 3.7. Photoelectrochemical Measurements

The photoelectrochemical measurements were conducted at a Zahner Zennium electrochemical workstation (Zennium, Zahner scientific instruments, Gundelsdorf, Germany) by a typical three-electrode system in 0.5 M Na_2_SO_4_. A graphite rod was used as a counter electrode, while an Ag/AgCl electrode was used as a reference electrode. The working electrode was prepared as follows, 5 mg of the catalyst was dispersed in 1 mL of ethanol for 1 h, and then 30 μL of Nafion (5 wt%) was added to the mixture, followed by ultrasonic dispersion for 20 min. Then, drop the obtained catalyst liquid onto the FTO, cover the electrode of 1 cm^2^, and dry for 2 h. Then, transient photocurrent plots and Mott-Schottky plots were obtained. Notably, the transient photocurrent plots were obtained under the same condition as photocatalytic CO_2_ reduction.

### 3.8. Photocatalytic Reduction of CO_2_

The photoreduction of CO_2_ by photocatalysts was conducted under visible light irradiation. In a typical procedure, 10 mg of photocatalysts were evenly dispersed on the quartz filter membrane, which had been placed in the solid-gas photocatalytic reaction device. Then, 2 mL of a mixture of acetonitrile and triethanolamine (the volume ratio of acetonitrile to TEOA is 4:1) was dropped on the sample. Afterwards, the system was degassed and filled with CO_2_ three times, making sure that all the air in the system was replaced by CO_2_. The system was kept at a dark condition for 1 h and then irradiated by 300 W Xe light with a 420 nm cutter to start the photoreduction reaction. The produced gas in the system was analyzed by a Gas Chromatography (GC7890, Agilent Technologies Co., Ltd., Santa Clara, CA, USA) with TCD and FID detector every 2 h.

## 4. Conclusions

In summary, GQDs had been in situ encapsulated into a highly stable porphyrin MOF, PCN-222, by the solvothermal method. The linker, deep purple porphyrins, makes GQDs@PCN-222 a highly visible light harvester up to 800 nm. After incorporation of GQDs, the shape of GQDs@PCN-222 particles remained, revealed by SEM. The crystallized and porous structure of obtained GQDs@PCN-222 also remained. As most of the GQDs were covered by thick PCN-222, it was hard to observe those GQDs using HRTEM directly, the treatment of digested GQDs@PCN-222 rods by immersion in a 1 mm aqueous KOH solution can make the incorporated GQDs visible in HRTEM. The GQDs@PCN-222 could combine the advantages of MOFs and GQDs, overcome the disadvantage of low catalytic activity of pure MOFs, and be used for high-efficiency photocatalytic CO_2_ reduction. Compared with PCN-222, the increased photocurrent and decreased PL emission of GQDs@PCN-222 indicated that the introduction of GQDs improves the separation efficiency and decreases the recombination of photogenerated electron–hole pairs of the catalyst, and then improves the visible light photocatalytic carbon dioxide reduction activity. Thus, the activity of GQDs@PCN-222 was almost four times higher than that of pure PCN-222, reaching 147.8 μmol/g/h in a 10 h period under visible light irradiation. This study demonstrated that the combination of GQDs and high light absorption MOFs provides a new platform for photocatalytic CO_2_ reduction.

## Data Availability

Not applicable.

## Supplementary Materials

The following supporting information can be downloaded at: https://www.mdpi.com/article/10.3390/molecules28124703/s1, References [38,47,51,53,61,62,63,64,65,66,67] are cited in the Supplementary Materials. Figure S1. Pore size distribution of PCN-222 and GQDs@PCN-222; Figure S2: SEM images of PCN-222; Figure S3: TEM and HRTEM images of GQDs/PCN-222; Figure S4: Solid UV-Vis diffuse reflectance spectrum and Tauc plot of H_2_TCPP; Figure S5: Solid UV-Vis diffuse reflectance spectrum (a) and Tauc plot (b) of PCN-222; Figure S6: Mott-Schottky plots of PCN-222 with a frequency of 500, 1000, and 1500 HZ; Figure S7: PXRD patterns of GQDs@PCN-222 before and after photocatalysis; Table S1: Comparison of photocatalysts for CO_2_ reduction with previous works.
