# Supplementary material for: In Situ Encapsulation of Graphene Quantum Dots in Highly Stable Porphyrin Metal-Organic Frameworks for Efficient Photocatalytic CO2 Reduction
Source: Molecules. 2023 Jun 12;28(12):4703. doi: 10.3390/molecules28124703 (PMC10303723; doi:10.3390/molecules28124703)
Supplement: Supplementary file 1 [file molecules-28-04703-s001.zip › molecules-2432196-supplementary.pdf]

In Situ Encapsulation of Graphene Quantum Dots in Highly Stable Porphyrin Metal-Organic Frameworks for Efficient Photocatalytic CO_2 Reduction

Qin Yu [1,†], Xusheng Wang [1,2,3,4,*,†], Wenbin Wu [1], Xinya Feng [1], Deyu Kong [1], Usman Khan [1], Xiaohui Ren [5] and Lan Li [6]

[1] Institute of Functional Porous Materials, School of Materials Science and Engineering, Zhejiang Sci-Tech University, Hangzhou 310018, China; 2021316101103@mails.zstu.edu.cn (Q.Y.); 2022316101108@mails.zstu.edu.cn (W.W.); 2022316101030@mails.zstu.edu.cn (X.F.); 2022316101070@mails.zstu.edu.cn (D.K.); usman@zstu.edu.cn (U.K.)
[2] Guangdong Provincial Key Laboratory of Functional Supramolecular Coordination Materials and Applications, Jinan University, Guangzhou 510632, China
[3] Tongxiang Research Institute, Zhejiang Sci-Tech University, Jiaxing 314500, China
[4] Zhejiang LINIX Motor Co., Ltd., Jinhua 322118, China
[5] The State Key Laboratory of Refractories and Metallurgy, School of Materials and Metallurgy, Wuhan University of Science and Technology, Wuhan 430081, China; xhren@wust.edu.cn
[6] College of Materials and Chemistry, China Jiliang University, Hangzhou 310018, China; lanli@cjlu.edu.cn
* Correspondence: xswang@zstu.edu.cn
† These authors contributed equally to this work.

Figures and tables

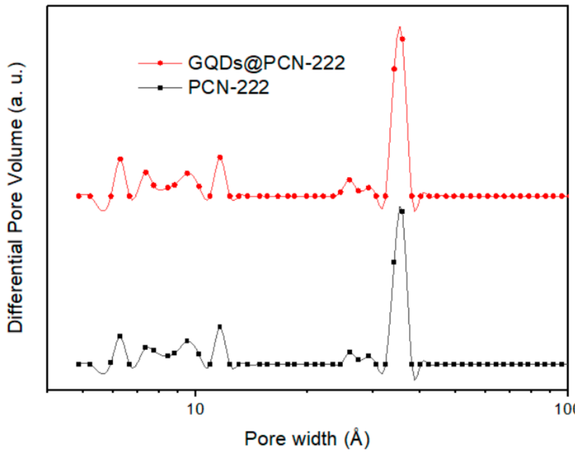

Figure S1. Pore size distribution of PCN-222 and GQDs@PCN-222.

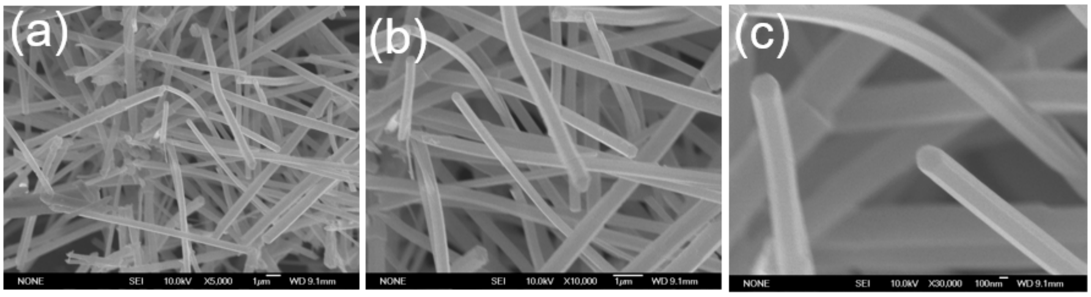

Figure S2. SEM images of PCN-222.

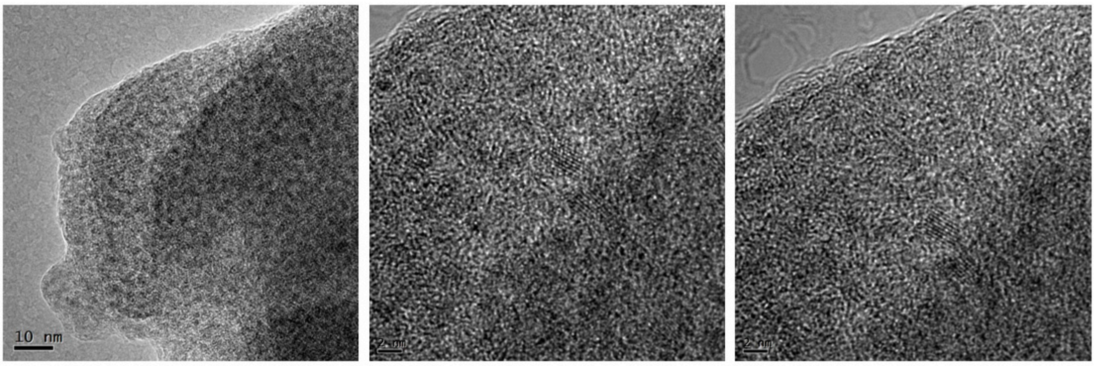

Figure S3. TEM and HRTEM images of GQDs/PCN-222.

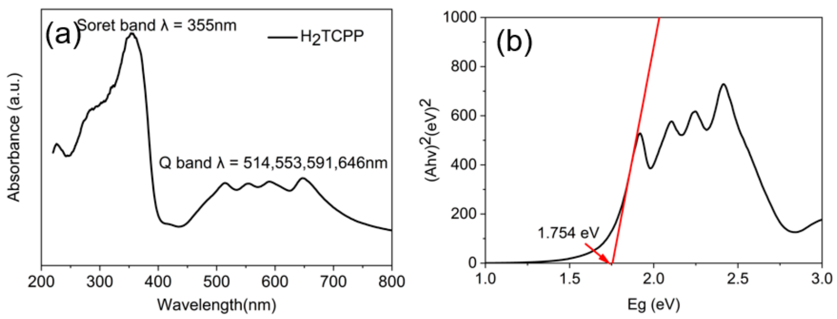

Figure S4. Solid UV-Vis diffuse reflectance spectrum and Tauc plot of H_2TCPP.

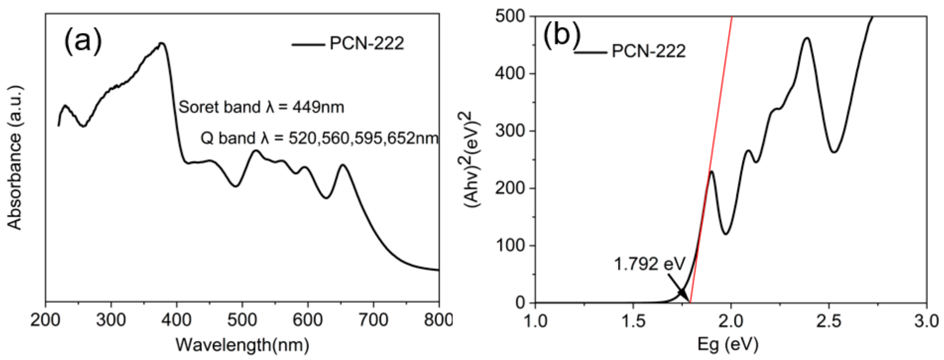

Figure S5. Solid UV-Vis diffuse reflectance spectrum (a) and Tauc plot (b) of PCN-222.

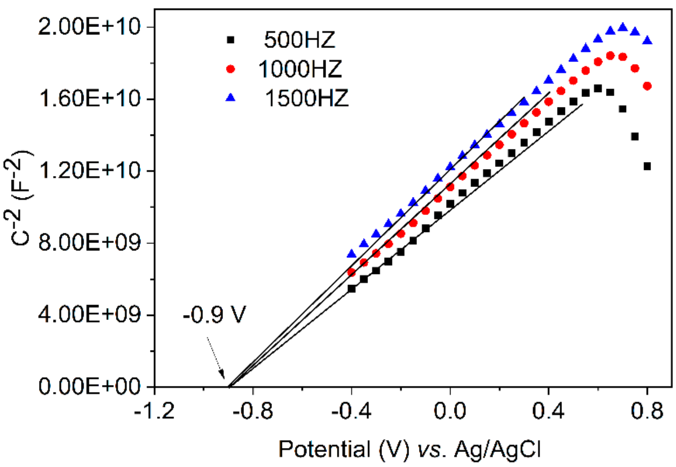

Figure S6. Mott-Schottky plots of PCN-222 with frequency of 500, 1000, and 1500 HZ.

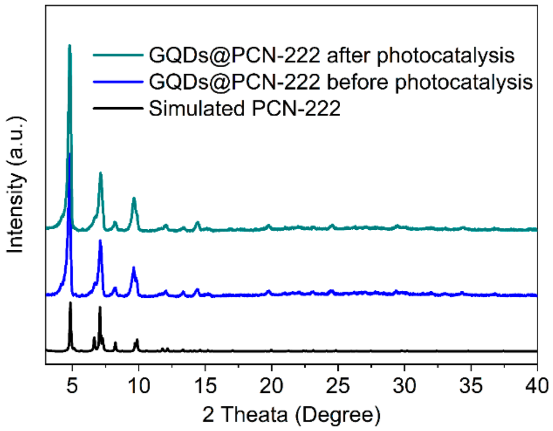

Figure S7. PXRD patterns of GQDs@PCN-222 before and after photocatalysis.

Table S1. Comparison of photocatalysts for CO_2 reduction with previous works.

Catalysts	Products and activity (μmol/g/h)	Reference
ZrPP-1-Co	CO: 14	[1]
NH_2-MIL-125(Ti)	$HCOO^-$: 16	[2]
MOF-525-Co	CO: 201.6<br>CH_4: 36.7	[3]
MOF-525-Zn	CO: 111.7<br>CH_4: 11.6	[3]
MOF-525	CO: 64.0<br>CH_4: 6.2	[3]
NNU-13	CH_4: 117<br>CO: 4	[4]
NNU-14	CH_4: 45<br>CO: 1	[4]
MIL-101(Cr)-EN	CO: 47.2	[5]
UiO-66/CNNS	CO: 9.8	[6]
$CsPbBr_3$@ZIF-8	CO: 0.5<br>CH_4: 1.8	[7]
$CsPbBr_3$@ZIF-67	CO: 0.8<br>CH_4: 3.5	[7]
Zn_2GeO_4/MgMOF-74	CO: 1.4	[8]

NCQDs/TiO_2	CO: 23<br>CH_4: 15	[9]
CQDs/Cu2O	CH_4: 4<br>Methanol: 68	[10]
CL@CQDs/Cu_2O	CH_4: 8<br>Methanol: 100	[10]
NH_2-MIL-88B(Fe)	CO: 12	[11]
GQD/NH_2-MIL-88B(Fe)	CO: 59	[11]
PCN-222	CO: 38	This work
GQDs@PCN-222	CO: 150	This work

Reference:

1. Chen, E. X.; Qiu, M.; Zhang, Y. F.; Zhu, Y. S.; Liu, L. Y.; Sun, Y. Y.; Bu, X.; Zhang, J.; Lin, Q. Acid and Base Resistant Zirconium Polyphenolate-Metalloporphyrin Scaffolds for Efficient CO_2 Photoreduction. *Adv. Mater.* **2018,** *30*, 1704388.
2. Fu, Y.; Sun, D.; Chen, Y.; Huang, R.; Ding, Z.; Fu, X.; Li, Z. An amine-functionalized titanium metal-organic framework photocatalyst with visible-light-induced activity for CO_2 reduction. *Angew. Chem. Int. Ed.* **2012,** *51*, 3364-3367.
3. Zhang, H.; Wei, J.; Dong, J.; Liu, G.; Shi, L.; An, P.; Zhao, G.; Kong, J.; Wang, X.; Meng, X.; Zhang, J.; Ye, J. Efficient Visible-Light-Driven Carbon Dioxide Reduction by a Single-Atom Implanted Metal-Organic Framework. *Angew. Chem. Int. Ed.* **2016,** *55*, 14310-14314.
4. Huang, Q.; Liu, J.; Feng, L.; Wang, Q.; Guan, W.; Dong, L. Z.; Zhang, L.; Yan, L. K.; Lan, Y. Q.; Zhou, H. C. Multielectron transportation of polyoxometalate-grafted metalloporphyrin coordination frameworks for selective CO2-to-CH4 photoconversion. *Natl. Sci. Rev.* **2020,** *7*, 53-63.
5. Xie, Y.; Fang, Z.; Li, L.; Yang, H.; Liu, T. F. Creating Chemisorption Sites for Enhanced CO2 Photoreduction Activity through Alkylamine Modification of MIL-101-Cr. *ACS Appl. Mater. Inter.* **2019,** *11*, 27017-27023.
6. Shi, L.; Wang, T.; Zhang, H.; Chang, K.; Ye, J. Electrostatic Self-Assembly of Nanosized Carbon Nitride Nanosheet onto a Zirconium Metal-Organic Framework for Enhanced Photocatalytic CO_2 Reduction. *Adv. Funct. Mater.* **2015,** *25*, 5360-5367.
7. Kong, Z.-C.; Liao, J.-F.; Dong, Y.-J.; Xu, Y.-F.; Chen, H.-Y.; Kuang, D.-B.; Su, C.-Y. Core@Shell CsPbBr3@Zeolitic Imidazolate Framework Nanocomposite for Efficient Photocatalytic CO2 Reduction. *ACS Energy Lett.* **2018,** *3*, 2656-2662.

8. Zhao, H.; Wang, X.; Feng, J.; Chen, Y.; Yang, X.; Gao, S.; Cao, R. Synthesis and characterization of Zn_2GeO_4/Mg-MOF-74 composites with enhanced photocatalytic activity for CO_2 reduction. *Catal. Sci. Technol.* **2018,** *8*, 1288-1295.
9. Li, M.; Wang, M.; Zhu, L.; Li, Y.; Yan, Z.; Shen, Z.; Cao, X. Facile microwave assisted synthesis of N-rich carbon quantum dots/dual-phase TiO2 heterostructured nanocomposites with high activity in CO2 photoreduction. *Appl. Catal. B: Environ.* **2018,** *231*, 269-276.
10. Li, H.; Deng, Y.; Liu, Y.; Zeng, X.; Wiley, D.; Huang, J. Carbon quantum dots and carbon layer double protected cuprous oxide for efficient visible light CO2 reduction. *Chem. Commun.* **2019,** *55*, 4419-4422.
11. Wang, X.; Yang, X.; Chen, C.; Li, H.; Huang, Y.; Cao, R. Graphene quantum dots supported on Fe-based metal-organic frameworks for efficient photocatalytic CO_2 reduction. *Acta Chim. Sinica* **2022,** *80*, 22-28.
